# Alleviating CB2-Dependent ER Stress and Mitochondrial Dysfunction Improves Chronic Cerebral Hypoperfusion-Induced Cognitive Impairment

**DOI:** 10.1007/s11481-024-10098-x

**Published:** 2024-01-12

**Authors:** Da Peng Wang, Kai Kang, Jian Hai, Qiao Li Lv, Zhe Bao Wu

**Affiliations:** 1 Department of Neurosurgery, Center of Pituitary Tumor, Ruijin Hospital, Shanghai Jiao Tong University School of Medicine, 197 Ruijin 2nd Road, Huangpu District, Shanghai, 200025 China; 2 Department of Neurosurgery, Tong Ji Hospital, School of Medicine, Tong Ji University, Shanghai, 200065 China; 3 School of Public Health, Fudan University, Shanghai, 200032 China; 4 Department of Research and Surveillance Evaluation, Shanghai Municipal Center for Health Promotion, Shanghai, 200040 China; 5 Jiangxi Key Laboratory of Translational Cancer Research, Jiangxi Cancer Hospital, Jiangxi, 330029 China; 6 Department of Neurosurgery, The First Affiliated Hospital of Wenzhou Medical University, Wenzhou, 325000 China

**Keywords:** Chronic cerebral hypoperfusion, Cognitive impairment, Endoplasmic reticulum stress, Endocannabinoid system, Fatty acid amide hydrolase, Mitochondria

## Abstract

Augmentation of endoplasmic reticulum (ER) stress may trigger excessive oxidative stress, which induces mitochondrial dysfunction. The fatty acid amide hydrolase inhibitor, URB597, shows anti-oxidation characteristics in multiple neurological disorders. The present study aimed to determine whether inhibition of ER stress was involved in the protective effects of URB597 against chronic cerebral hypoperfusion (CCH)-induced cognitive impairment. Hippocampal HT-22 cells were exposed to oxygen-glucose deprivation. The cell viability, apoptosis, ER stress, mitochondrial ATP, and oxidative stress levels were assessed following treatment with URB597, benzenebutyric acid (4-PBA), and thapsigargin (TG). Furthermore, the effects of URB597 on ER stress and related pathways were investigated in the CCH animal model, including Morris water maze testing of cognition, western blotting analysis of ER stress signaling, and transmission electron microscopy of mitochondrial and ER ultrastructure changes. The results suggested that cerebral ischemia caused ER stress with upregulation of ER stress signaling-related proteins, mitochondrial dysfunction, neuronal apoptosis, ultrastructural injuries of mitochondria-associated ER membranes, and cognitive decline. Co-immunoprecipitation experiments confirmed the interaction between CB2 and β-Arrestin1. Inhibiting ER stress by URB597 improved these changes by activating CB2/β-Arrestin1 signaling, which was reversed by the CB2 antagonist, AM630. Together, the results identified a novel mechanism of URB597, involving CCH-induced cognitive impairment alleviation of CB2-dependent ER stress and mitochondrial dysfunction. Furthermore, this study identified CB2 as a potential target for therapy of ischemic cerebrovascular diseases.

**Graphical Abstract:**

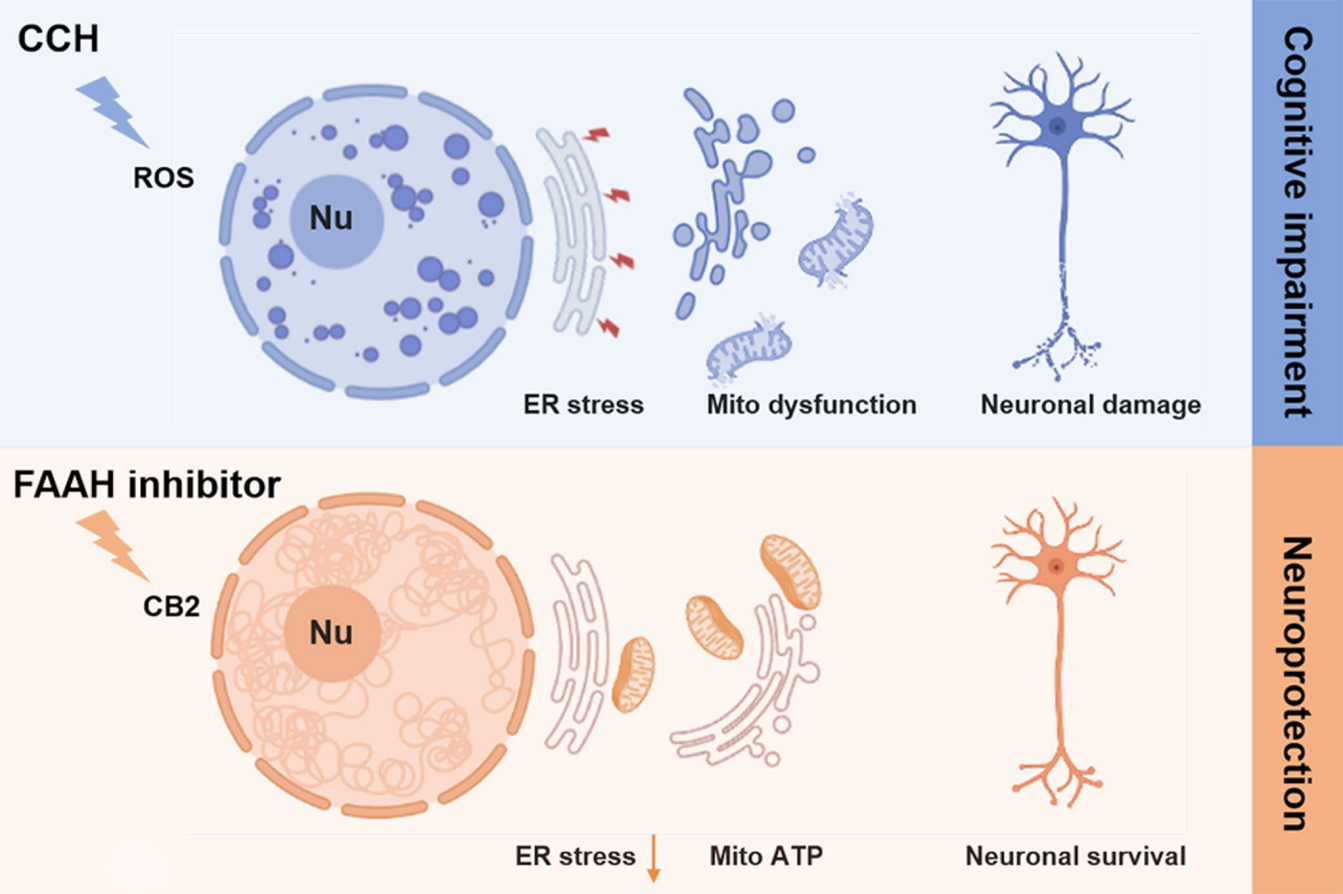

## Introduction

The morbidity of ischemic cerebrovascular disease increases with age; the elderly over 65-years-old are very prone to cerebral ischemia cognitive decline (Rajeev et al. 2022). Chronic cerebral hypoperfusion (CCH), a hemodynamic feature of ischemic cerebrovascular diseases, including carotid stenosis, atherosclerosis of large or small cerebral vessels, and other cerebral-vascular or senile diseases, is a major cause of cognitive impairment and vascular dementia (VaD) (Ciacciarelli et al. 2020). VaD is the second most common cause of dementia, accounting for approximately 15 − 20% of dementia in North America and Europe, and roughly 30% in Asia (Chan et al. 2013; Wolters and Ikram 2019), which results in a heavy burden to worldwide public health. Because of a lack of unified diagnostic criteria and effective intervention targets, progress in its treatment has been difficult (O’Brien and Thomas 2015). Long-term insufficient cerebral blood flow can activate inflammation, oxidative stress, and neurodegeneration, leading to multiple neurocyte injuries(Kalaria 2018). These cellular injury cascades may trigger or exacerbate homeostatic disturbances between or within cells and organelles, causing subcellular deteriorations and irreversible hippocampal neuronal damage (Rajeev et al. 2023). Therefore, additional effort is still needed to identify the mechanism of CCH-induced memory dysfunction, which will hopefully identify specific intervention targets.

The endocannabinoid system (ECS) is composed of cannabinoid receptors (CBR1 and CBR2), endogenous ligands-anandamides, 2-arachidonic glycerol, and endogenous degrading enzymes (Lu and Mackie 2021). The ECS involves the regulation of numerous pathophysiological processes, such as habituation, neuropathic pain, neuroinflammation, and cognitive deficits (Cristino et al. 2020). The 3´-carbamoylbiphenyl-3-yl cyclohexyl carbamate (URB597), a fatty acid amide hydrolase (FAAH) inhibitor, mediates ECS bioactivities and levels, providing beneficial effects during neurodegeneration, ischemia-reperfusion injury, and oxidative stress (Wang et al. 2021b, 2022; Cakir et al. 2023). Mitochondria and the endoplasmic reticulum (ER) are essential organelles that produce reactive oxygen species (ROS) in response to neuronal oxidative stress during cerebral ischemia (Singh-Mallah et al. 2019). Inhibition of excessive hippocampal mitochondria ROS production and mitophagy by URB597 targeting CCH-induced neuroinflammatory responses have been previously reported (Wang et al. 2017b; Su et al. 2018). In contrast, some reports have suggested that URB597 impaired long-term potentiation, learning, and memory (Basavarajappa et al. 2014). Increasing studies have shown that ECS regulated brain energy metabolism, hippocampal neurogenesis, synaptic plasticity, and organogenesis (Forte et al. 2021). More importantly, the effects of URB597 on mitochondria and the ER, as well as the potential molecular mechanisms of neuro-modulation during CCH have not yet been systematically assessed.

To address these issues, in the present study, we determined the effects of URB597 on ER stress and mitochondrial function, as well as the underlying mechanisms associated with its therapeutic potential in brain ischemia treatment.

## Materials and Methods

### Materials and Reagents

The FAAH inhibitor (URB597) (No. HY-10,864), the ER stress inhibitor and inducer, [benzenebutyric acid (4-PBA) (No. HY-A0281) and thapsigargin (TG) (No. HY-13,433), respectively], and a selective CB2 antagonist (6-lodopravadoline, AM630) (No. HY-15,421) were all purchased from the Med Chem Express (MCE, Shanghai, China). The drugs were dissolved using 10% dimethyl sulfoxide, 40% polyethylene glycol-300, 5% Tween-80, and 45% saline according to the manufacturer’s instructions for in vivo experiments. The doses of agents were selected as previously reported (Wang et al. 2017a, 2022; Reddy et al. 2019; Pawar et al. 2022). Briefly, URB597 at a concentration of 2 µM for cell experiments, was administered via intraperitoneal (i.p.) injection at 0.3 mg/kg/day for animal experiments, 4-PBA at a concentration of 4 µM for cell experiments, was administered i.p. to rats at 40 mg/kg/day, and TG was used at a concentration of 0.02 µM for cell experiments. Rabbit monoclonal/polyclonal antibodies against NeuN (24307T), glial fibrillary acidic protein (GFAP, 80788T), caspase-9 (9508T), β-tubulin (2146 S), and phospho-PERK (Thr980, 3179 S) were from Cell Signaling Technology (Danvers, MA, USA). Antibodies against 78-kDa glucose-regulated protein (GRP78) (ab212054), protein kinase R-like ER kinase (PERK) (ab229912), CB2 (ab35601), and GAPDH (ab8245) were from Abcam (Cambridge, MA, USA). Antibodies to Iba-1(DF6642) and C/EBP-homologous protein (CHOP, DF6025) were from Affinity (Cincinnati, OH, USA). Antibody to translocase of the outer mitochondrial membrane 20 (TOMM20) (WH0009804M1) was from Sigma-Aldrich (St. Louis, MO, USA). Antibodies to β-Arrestin-1 (sc-53,780) and cytochrome-c (Cyt-c, sc-13,156) were from Santa Cruz Biotechnology (Shanghai, China). The Alexa 488/594-conjugated goat anti-rabbit/mouse antibody was from Invitrogen (Carlsbad, CA, USA). The antibody to ATF6 (AF6243), the enhanced BCA protein assay kit (P0010), the Annexin V-FITC apoptosis detection kit (C1062), the ATP assay kit (S0026), the total superoxide dismutase (SOD) assay kit (S0109), the catalase assay kit (CAT, S0051), the lipid peroxidation malondialdehyde (MDA) assay kit (MDA, S0131S), and the dihydroethidium (DHE, S0063) and Nissl staining solution (C0117) were from Beyotime Biotechnology (Shanghai, China).

### The CCH Model and Treatment Groups

Sprague-Dawley male rats (1-month-old, 150 − 180 g) were from the experimental animal center of Shanghai Sippr-BK Laboratory Animals (Shanghai, China). They were housed in a SPF animal center with a room temperature of 24 °C and 60% humidity, with free access to food and water during a 12 h light/dark cycle. CCH was induced by bilateral common carotid artery occlusion (BCCAO) as described in our previous studies (Wang et al. 2017a, b). After 2 weeks of acclimatization, the rats (age, 6-weeks-old; body weight, approximately 200 g) were initially anesthetized with 5% isoflurane in 70% nitrogen and 30% oxygen, then maintained using 2% isoflurane in 0.5 L/min oxygen. A midline cervical incision was performed to expose the bilateral common carotid arteries, which were carefully double-ligated with 5 − 0 silk sutures. Sham-operated animals were not subjected to carotid artery ligation.

CCH rats were then randomly divided into five treatment groups: (1) the BCCAO group (M), (2) the BCCAO + URB597 group (MU), (3) the BCCAO + 4-PBA group (MP), (4) the BCCAO + URB597 + 4-PBA group (MUP), and (5) the BCCAO + URB597 + 4-PBA + AM630 group (MUPA) (*n* = 8 in each group). Four rats were dead after BCCAO surgery. Rats received daily injections of URB597 (0.3 mg/kg/day, i.p.), 4-PBA (40 mg/kg/day, i.p.), and AM630 (1 mg/kg/day, i.p.) for 4 weeks in the MU, MP, MUP, MUPA groups, respectively. Rats in the Sham and M groups (*n* = 8 in each group) received daily injections of an equal amount of vehicle. Rats were decapitated 2 h after the last injection and the brains were immediately removed for experiments, or stored at -80 °C.

### The Oxygen-Glucose Deprivation (OGD) Model and Treatment Groups

The mouse hippocampal neuronal HT22 cell line was purchased from a public cell bank (ATCC, Manassas, VA, USA). The cells were cultured in Dulbecco’s Modified Eagle Medium (DMEM) (Beyotime Biotechnology) supplemented with 10% fetal bovine serum (HyClone, Ogden, UT, USA) and 1% penicillin/streptomycin (HyClone) in an incubator (Heraeus, Hanau, Germany) at 37 °C in 5% CO_2_. For OGD, HT22 cells were seeded in 96-well plates at a density of 1 × 10^5^ cells/mL and were cultured in glucose-free DMEM at 37 °C in 0.5% O_2_, 94.5% N_2_, and 5% CO_2_ for 4 h. The cells were then incubated in a maintenance medium for 24 h under normal conditions before subsequent experiments.

HT22 cells in the control group were treated identically except that they were not exposed to OGD. Experimental treatment groups were as follows: (1) the control group (Con), (2) the OGD group (OGD), (3) the OGD + 4-PBA (4 µM) treatment group (4-PBA), (4) the OGD + URB597 (2 µM) treatment group (URB), and (6) the OGD + TG (0.02 µM) treatment group (TG).

### Cell Viability Assay

Cell viability was assessed using the 3-(4,5-dimethyl-2-thiazolyl)-2,5-diphenyl-2-H-tetrazolium bromide (MTT) assay as previously reported (Chang et al. 2021; Wang et al. 2022). A solution with 20 µL MTT [5 mg/mL MTT in phosphate-buffered saline (PBS), pH 7.4] was added to each group. Then, neurons were incubated for 4 h at 37 °C in 5% CO_2_. The absorbance (OD) was measured spectrophotometrically at 490 nm on a microplate reader (Epoch; Bio-Tek, Winooski, VT USA).

### Measurement of Oxidative Stress and ATP

Oxidative stress and ATP levels were evaluated by determination of SOD, CAT, MDA, and ATP using commercial kits. All assays were performed using a microplate reader according to the manufacturer’s instructions (manufacturer and address) (Wang et al. 2020a).

### DHE Staining

The intracellular ROS were detected using DHE staining. Cells were plated in 24-well plates, then fixed with formaldehyde for 30 min, stained with 30 µM DHE staining at room temperature for 5 min, then checked with an immunofluorescence assay using ImageJ software (Version 1.46r; National Institutes of Health, Bethesda, MD, USA) (Gao et al. 2019).

### Annexin-V-FITC Flow Cytometry Analysis

Cell apoptosis was quantified using an Annexin V Apoptosis Detection kit according to the manufacturer’s instructions (manufacturer and address). Briefly, 10 µL propidium iodide and 5 µL Annexin-V-FITC solution were added to HT22 cells, followed by incubation for 15 min in the dark at room temperature. Finally, the cells were collected into flow cytometry tubes, and cell apoptosis was measured using a flow cytometer at 488 nm.

### Nissl Staining

Coronal slices (10 μm thick) were used to estimate hippocampal neural tissue damage using Nissl staining as earlier reported (Xu et al. 2018). Briefly, the coronal cryosections of the brain were stained with 0.75% cresyl violet, dehydrated using graded alcohol percentages (70%, 95%, and 100%), and placed in xylene. The slices (*n* = 3) were visualized using a microscope (BX53; Olympus, Tokyo, Japan) at ×200 by an investigator blinded to the identities of the treatments.

### Morris Water Maze (MWM)

The MWM was used to measure hippocampus-dependent learning and memory, as previously described (Wang et al. 2017a, 2021c). Rats were tested in a circular tank, 1.5 m in diameter with a platform of 14 cm in diameter below the water surface (1.5 cm). Animals were trained for 4 days with four trials per day by looking at the platform in the tank with water (25 ± 1 °C) (*n* = 8 rats per group). On day 5, the platform was removed and rats were allowed to swim freely for 60 s in the probe trial. A video camera and Human Visual System Image Software (HVS Image, Hampton, UK) were used to observe and record the times spent in the target quadrant, platform position crossings, and the swimming pattern of each rat.

### Transmission Electron Microscopy (TEM)

Brains were prepared for TEM analysis to determine ultrastructural changes, using previously reported procedures (Wang et al. 2017a, b). Briefly, tissues were dehydrated by alcohol and embedded with a mixture of acetone and ethoxylate resin (Ladd Research Industries, Burlington, VT). Brain sections, cut to a thickness of 600 nm using the LKB Huxley ultramicrotome, were placed on copper grids, then stained with uranyl acetate and lead citrate (Wang et al. 2017b). Finally, the ultrastructure changes of organelles in the hippocampus CA1 area were observed using a transmission electron microscope (CM-120; Philips, Amsterdam, The Netherlands). The degree of mitochondrial damage and the ultrastructure changes of mitochondria-associated ER membranes (MAMs) were semi-quantitatively analyzed by one investigator, blinded to the identities of the treatment groups, according to published guidelines as shown in Table 1 (Flameng’s score) (Flameng et al. 1980; Paillusson et al. 2017; Ouyang et al. 2022).

**Table 1:** The criteria for scoring the neuronal mitochondria injury using TEM (Flameng et al. 1980)

Scores	Injurious manifestations and ultrastructural changes
0	Normal structure
1	The structure is basically normal, but the matrix particles are lost (slight swelling, matrix density is reduced, cristae separation)
2	Mitochondrial swelling (reduced matrix density, cristae separation), the matrix is transparent; cristae are not broken
3	Mitochondrial cristae rupture, matrix coagulation (severe swelling)
4	Mitochondrial cristae rupture, the integrity of the inner and outer membranes disappear and become vacuolated (severe swelling, rupture of inner and outer membranes)

### Immunofluorescence Staining

Brain sections or HT22 cells were fixed in precooled 4% paraformaldehyde for 20 min, permeabilized with 0.1% Triton X-100 (Sigma-Aldrich) for 30 min, then blocked with 5% bovine serum albumin (BSA) in PBS for 30 min at room temperature. The fixed tissues and cells were incubated with primary antibodies against GRP78 (1:300), TOMM20 (1:300), NeuN (1:300), Iba-1 (1:300), or GFAP (1:300) in 5% BSA overnight at 4 °C(Jin et al. 2018; Wang et al. 2022). Subsequently, samples were counterstained with 4´,6-diamidino-2-phenylindole after incubating with deconjugated secondary antibodies for 2 h in the dark. A fluorescence microscope (Zeiss, Jena, Germany) was used to photograph the immunofluorescent images. Finally, three fields of view in each group were used to estimate the mean fluorescence intensity, by an investigator blinded to the identities of the groups.

### Western Blotting and Immunoprecipitation

Proteins from neuronal tissues in the hippocampus CA1 area were extracted and quantified using a total protein extraction kit (BC3711; Solarbio, Beijing, China) and a BCA protein assay kit (P0012S; Beyotime) according to the manufacturer’s protocols. Equal amounts of protein were separated by SDS-PAGE on a 6 − 12% polyacrylamide gel, then transferred onto polyvinylidene difluoride (PVDF) membranes. The PVDF membranes were subsequently probed with primary antibodies against the following proteins overnight at 4℃: GRP78 (1:1,000), ATF6 (1:500), PERK (1:1,000), p-PERK (1:1,000), CB2 (1:500), β-Arrestin-1(1:500), CHOP (1:500), Cyt-c (1:500), caspase-9 (1:1,000), GAPDH (1:5,000), and β-tubulin (1:5,000) used as an internal loading control. Following washing in PBS, the membranes were incubated with secondary antibodies conjugated with horseradish peroxidase, at room temperature for 1 h. The western blot protein bands were visualized using the enhanced chemiluminescence system (Millipore, Watford, UK) and quantified by ImageJ software.

For immunoprecipitation, 300 µg of protein extract was incubated with the antibodies against CB2, β-Arrestin-1, or unspecific IgG at 4 °C overnight, and protein-A/G agarose was added for another 2–3 h at 4 °C (Wang et al. 2021a). The immune precipitates were centrifuged, washed, suspended, and subjected to western blotting analysis.

### Statistical Analysis

Each experiment was conducted at least in triplicate. The data are presented as the mean ± standard deviation (SD) and were analyzed by SPSS statistical software for Windows, version 20.0 (SPSS, Chicago, IL, USA). The repeated-measures mixed analyses of variance (ANOVA) with Tukey post-hoc test was used to assess group and training day differences. One-way analysis of variance, followed by the Tukey post-hoc test was conducted to evaluate statistical differences among the experimental groups. Significance was defined as *P* < 0.05.

## Results

### The FAAH Inhibitor, URB597, Promotes Neuronal Survival in OGD in Hippocampal HT22 Cells

**Fig. 1:**
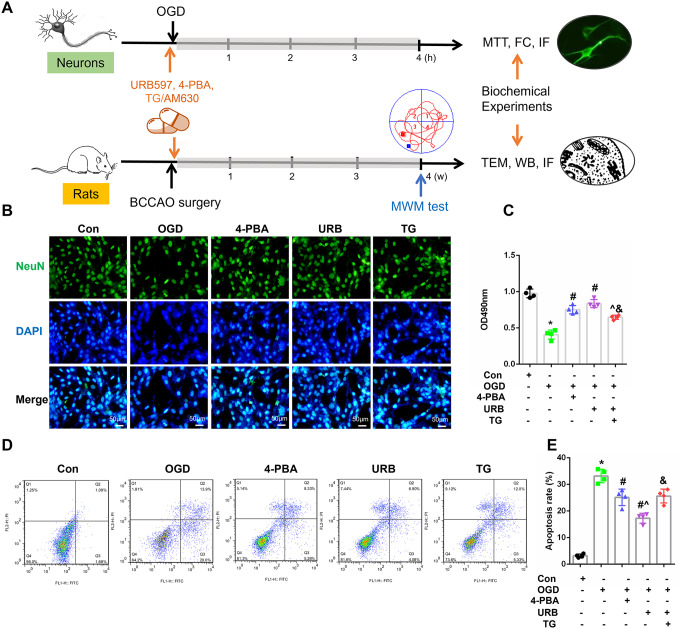
Effects of URB597, 4-PBA, and TG on HT22 cell survival and apoptosis in OGD. **A** An experimental flow chart. **B** Representative NeuN (green) immunofluorescence for HT22 neuronal cells. **C** Statistical analysis of the cell viability using the MTT assay. **D**, **E** Cellular apoptosis was detected by Annexin V-FITC/propidium iodide flow cytometry analysis. ^∗^*p* < 0.05 νs. Con, ^#^*p* < 0.05 νs. OGD, ^^^*p* < 0.05 νs. 4-PBA, ^&^*p* < 0.05 νs. URB597, (*n* = 4). Magnification: 200×, scale bar: 50 μm

The flow chart for the experimental procedure is shown in Fig. 1A. ER stress can be triggered by cerebral ischemia, leading to irreversible neuronal damage. Neuronal survival and apoptosis were estimated using MTT and flow cytometry analyses, respectively (Fig. 1B, D). Here, we found that 4-PBA and URB597 improved the survival stage of HT22 neuronal cells, and reduced cell apoptosis following OGD treatment (Fig. 1C, E, all, *P* < 0.05 vs. the Con group). Compared with individual intervention with URB597, apoptosis was significantly increased after adding the ER stress agonist, TG (Fig. 1E, *P* < 0.05 vs. the URB group), which suggested that activating ERS aggravated neuronal injury. Thus, these results suggested the neuroprotection of URB597 against OGD-induced neuronal apoptosis was associated with suppressing ERS.

### The FAAH Inhibitor, URB597, Inhibits OGD-Induced ERS in Hippocampal HT22 Cells

**Fig. 2:**
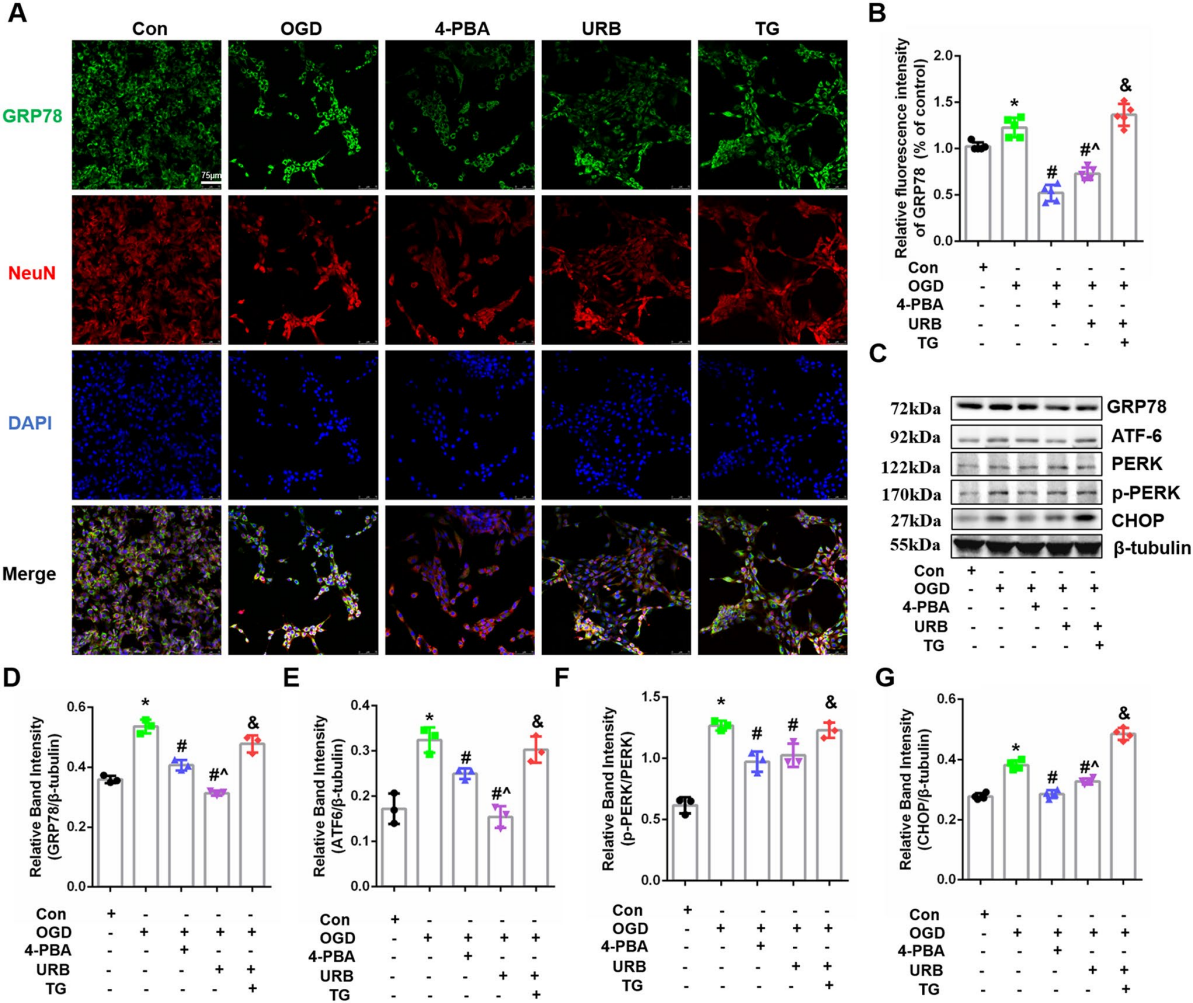
Effects of URB597, 4-PBA, and TG on HT22 cell ER stress in OGD. **A** Representative GRP78 (green) and NeuN (red) immunofluorescence. **B** Statistical analysis of the fluorescence intensity of GRP78. **C** The expression of ER stress signaling-related proteins, including GRP78, ATF-6, p-PERK/PERK, and CHOP. **D**–**G** Expression histograms. ^∗^*p* < 0.05 νs. Con, ^#^*p* < 0.05 νs. OGD, ^^^*p* < 0.05 νs. 4-PBA, ^&^*p* < 0.05 νs. URB597, (*n* = 3)

As shown in Fig. 2, ERS-related GRP78 labeling with green fluorescence was very significant in the OGD group (*P* < 0.05 vs. Con). While the green intensity was reduced by 4-PBA and URB597 treatments (*P* < 0.05 vs. OGD), this inhibition of GRP78 fluorescence with URB597 was reversed by TG (*P* < 0.05 vs. URB597) (Fig. 2A, B). Furthermore, the presence of ERS pathway-related proteins, ATF6, PERK, p-PERK, and CHOP, were determined using western blotting (Fig. 2C). The results showed that GRP78, ATF6, PERK, p-PERK, and CHOP levels significantly increased following OGD treatment, relative to the control treatment, whereas 4-PBA and URB597 treatments reduced the expressions of these proteins (all, *P* < 0.05 vs. OGD group). When compared with the URB59 group, TG treatment upregulated the expressions of GRP78, ATF6, PERK, p-PERK, and CHOP (Fig. 2D–G), showing that URB597 suppressed the OGD-induced ERS and ER-related apoptosis of CHOP.

### The FAAH Inhibitor, URB597, Alleviates OGD-Induced Mitochondrial Injury in Hippocampal HT22 Cells

**Fig. 3:**
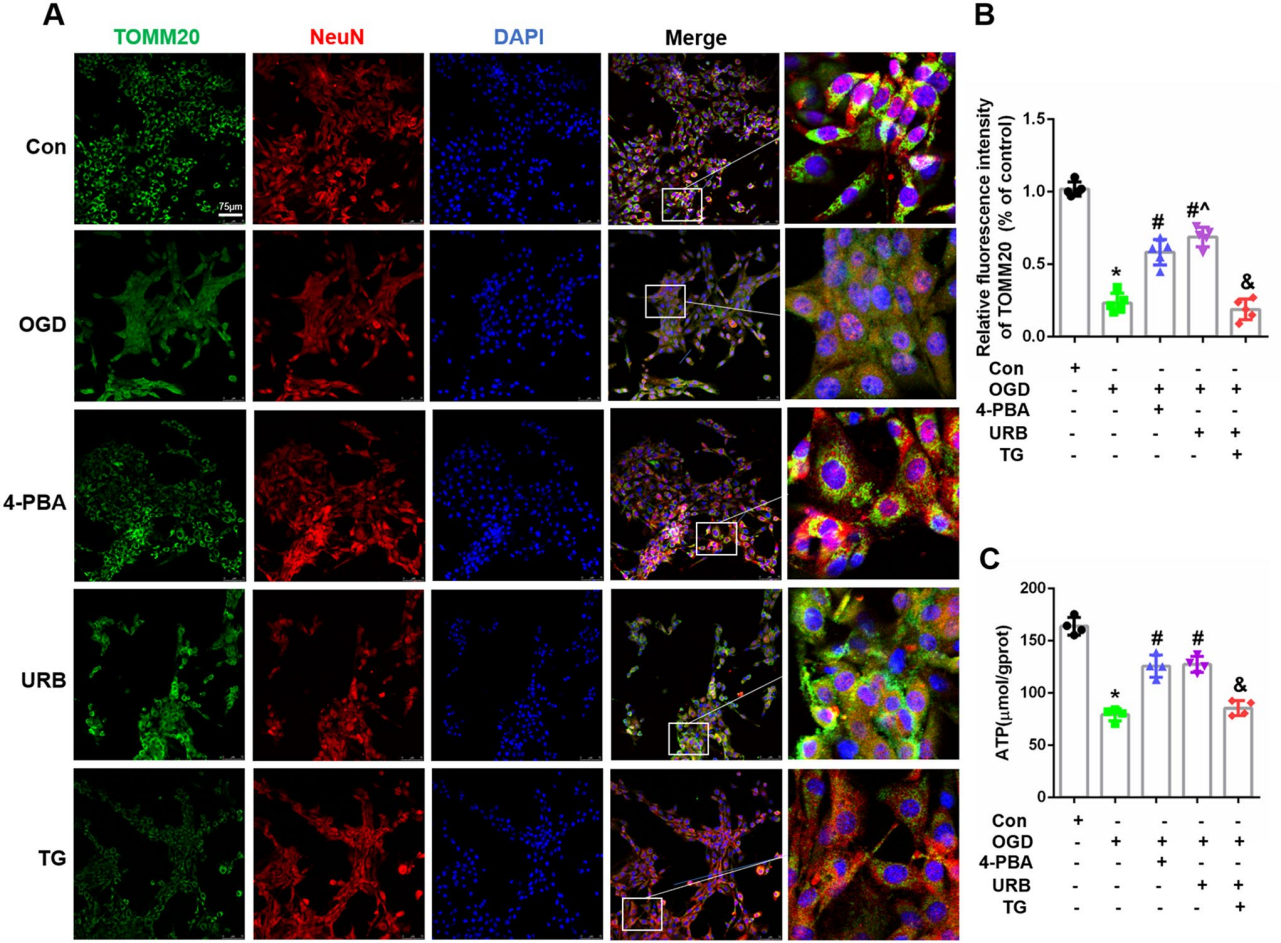
Effects of URB597, 4-PBA, and TG on HT22 cell mitochondrial function in OGD. **A** Representative TOMM20 (green) and NeuN (red) immunofluorescence. **B** Statistical analysis of fluorescence intensity of TOMM20. **C** Statistical analysis of ATP levels. ^∗^*p* < 0.05 νs. Con, ^#^*p* < 0.05 νs. OGD, ^^^*p* < 0.05 νs. 4-PBA, ^&^*p* < 0.05 νs. URB597, (*n* = 4)

We further evaluated the effects of these three reagents on mitochondrial structure and function. Double staining with NeuN (red) and TOMM20 (a mitochondrial marker, green) revealed that green fluorescence intensity was enhanced by 4-PBA and URB597 treatment, but not by TG (all, *P* < 0.05 vs. OGD) (Fig. 3A, B). The relative fluorescence intensity of TOMM20 decreased after co-treatment with URB597 and TG (*P* < 0.05 vs. URB597), while ATP, 4-PBA, and URB597 treatments promoted ATP production (*P* < 0.05 vs. OGD). However, treatment with the ERS agonist, TG, resulted in a reduction in ATP production. Together, these results confirmed that inhibition of ERS by 4-PBA as well as by URB597 alleviated OGD-induced mitochondrial injury.

### The FAAH Inhibitor, URB597, Suppresses OGD-Induced ER and Mitochondrial Stress in Hippocampal HT22 Cells

**Fig. 4:**
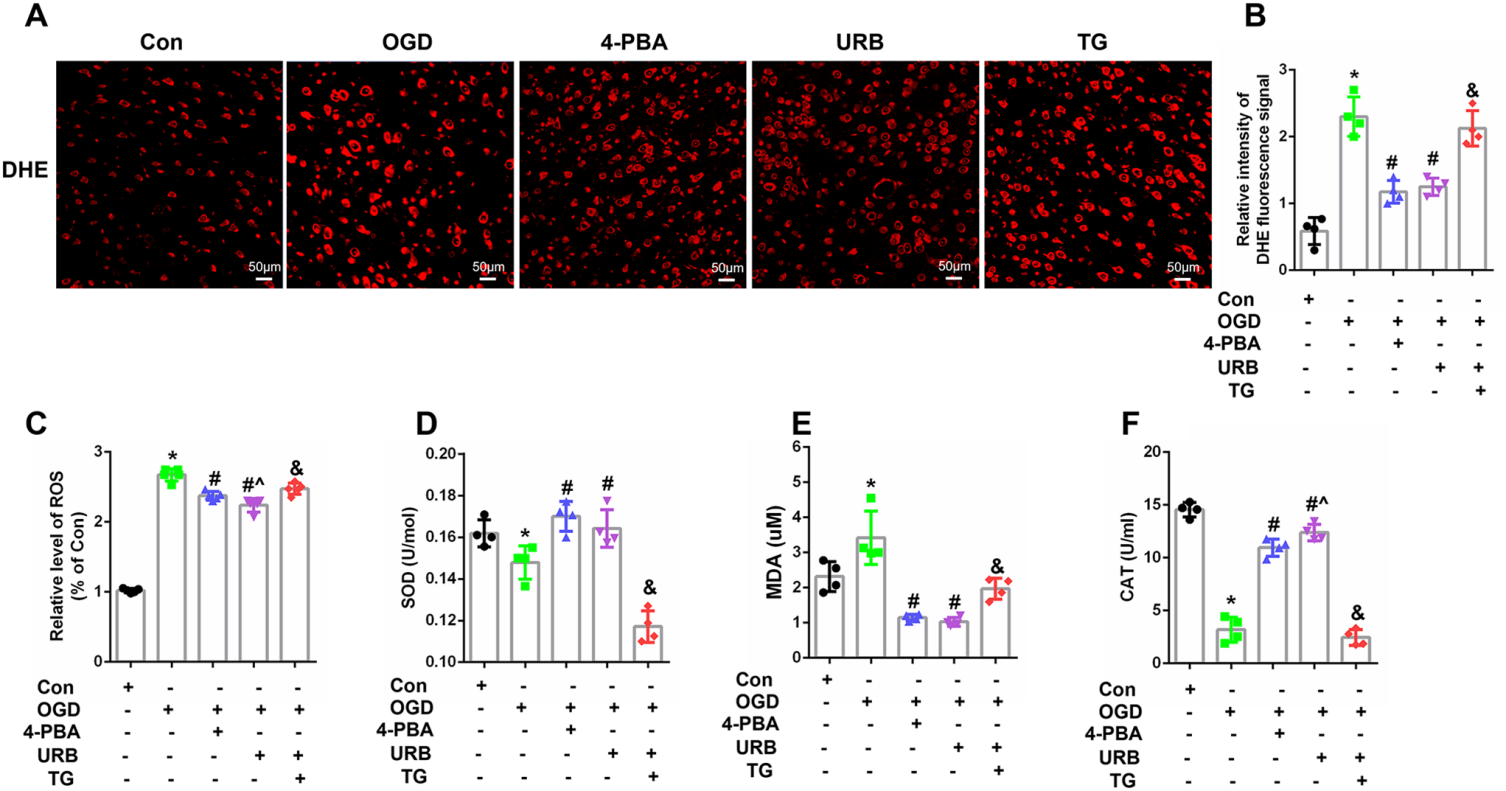
Effects of URB597, 4-PBA, and TG on ER and mitochondrial oxidative stress in OGD. **A** Immunofluorescent DHE staining of reactive oxygen species (ROS) levels. **B** Quantitative analysis of the DHE signal. **C**–**F** Quantification of ROS, SOD, CAT, and MDA levels in HT22 cells. ^∗^*p* < 0.05 νs. Con, ^#^*p* < 0.05 νs. OGD, ^^^*p* < 0.05 νs. 4-PBA, ^&^*p* < 0.05 νs. URB597, (*n* = 4). Magnification: 200×, scale bar: 50 μm

Mitochondria and the ER are the main organelles that produce ROS. The imbalance between oxidation and antioxidant products causes an oxidative stress response. Various biomarkers, such as ROS, SOD, MDA, and CAT were used to assess the level of oxidative stress in hippocampal HT22 neuronal cells. Immunofluorescent DHE (red fluorescence, Fig. 4A) staining of ROS levels was increased after OGD treatment (*P* < 0.05 vs. Con), and the red fluorescence was partially downregulated in the 4-PBA and URB597 treatment groups, when compared with the OGD group (*P* < 0.05 vs. OGD, Fig. 4B), indicating URB597 mitigated the ROS levels in OGD neurons. Furthermore, cells in the OGD group showed decreased levels of SOD and CAT (*P* < 0.05, respectively, vs. Con) and increased levels of ROS and MDA (*P* < 0.05, vs. Con). URB597 treatment reversed all of these effects (Fig. 4C–F). TG weakened the antioxidant effects of URB597 in OGD (*p* < 0.05 vs. URB597, Fig. 4C–F). Together, these results showed that URB597 inhibited OGD-induced ROS production and the resultant oxidative stress response.

### The FAAH Inhibitor, URB597, Improves CCH-Induced Cognitive Deficits

**Fig. 5:**
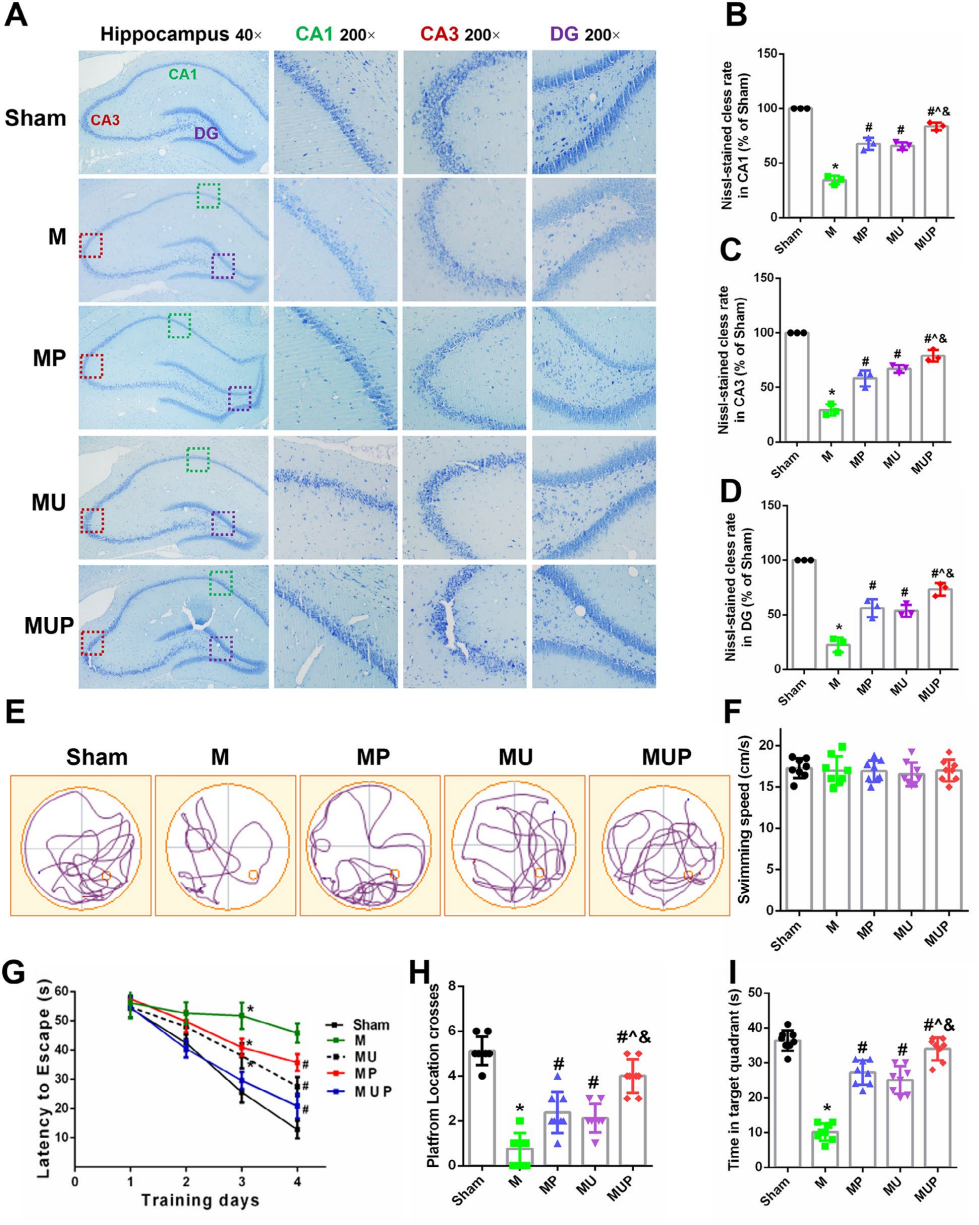
Effects of URB597 and 4-PBA on CCH-induced hippocampal neuronal injury and spatial learning and memory deficits. **A** Representative Nissl staining in hippocampal CA1, CA3, and DG areas. **B**–**D** Quantitative analysis of Nissl-stained cells rate in CA1, CA3, and DG, respectively (*n* = 3). **E** Representative swimming paths of rats in the probe trial. **F** The swimming speed during the probe trial. **G** Escape latencies during the training trials **H**, **I** The time spent in the target quadrant and the platform location crosses from different groups during the probe trial. ^*^p < 0.05 vs. Sham; ^#^*p* < 0.05 νs. M, ^*p* < 0.05 νs. MP, ^&^*p* < 0.05 νs. MU, (*n* = 8)

We further assessed the effects of URB597 on hippocampal neurons and cognitive ability using Nissl staining and the MWM test, respectively. As shown in Fig. 5A, many neurons in the CA1, CA3, and DG regions of the hippocampus in mice of the M group became shrunken with lighter Nissl staining, compared with those in the Sham group (*P* < 0.05 vs. Sham) (Fig. 5A–D). The reduced Nissl staining of bodies was increased after URB597 treatment, 4-PBA treatment, and co-treatment (*P* < 0.05 vs. M) (Fig. 5B–D), indicating that URB597 and 4-PBA treatments prevented structural damage of neurons in hippocampal regions of mice induced by CCH. In the visible platform trial, the escape latency of CCH mice was significantly improved following URB597 treatment, 4-PBA treatment, and co-treatment, without differences in swimming speeds (Fig. 5E–F). In the spatial probe trial, the crossed number of platforms and the time spent in the target quadrant in the M group were significantly lower than in mice in the Sham group (*P* < 0.05 vs. Sham) (Fig. 5G–I). However, the number of crossings and the finding times partially increased in mice in the URB597 and 4-PBA treatment groups, when compared with the M group (all, *P* < 0.05 vs. M). Moreover, co-treatment had optimal effectiveness in learning and memory (*P* < 0.05 vs. MUP) (Fig. 5H–I). Collectively, these results suggested that URB597 and 4-PBA minimized CCH-induced cognitive impairment in mice.

### The FAAH Inhibitor URB597 Alleviates CCH-Induced ER Stress and Mitochondrial Dysfunction

**Fig. 6:**
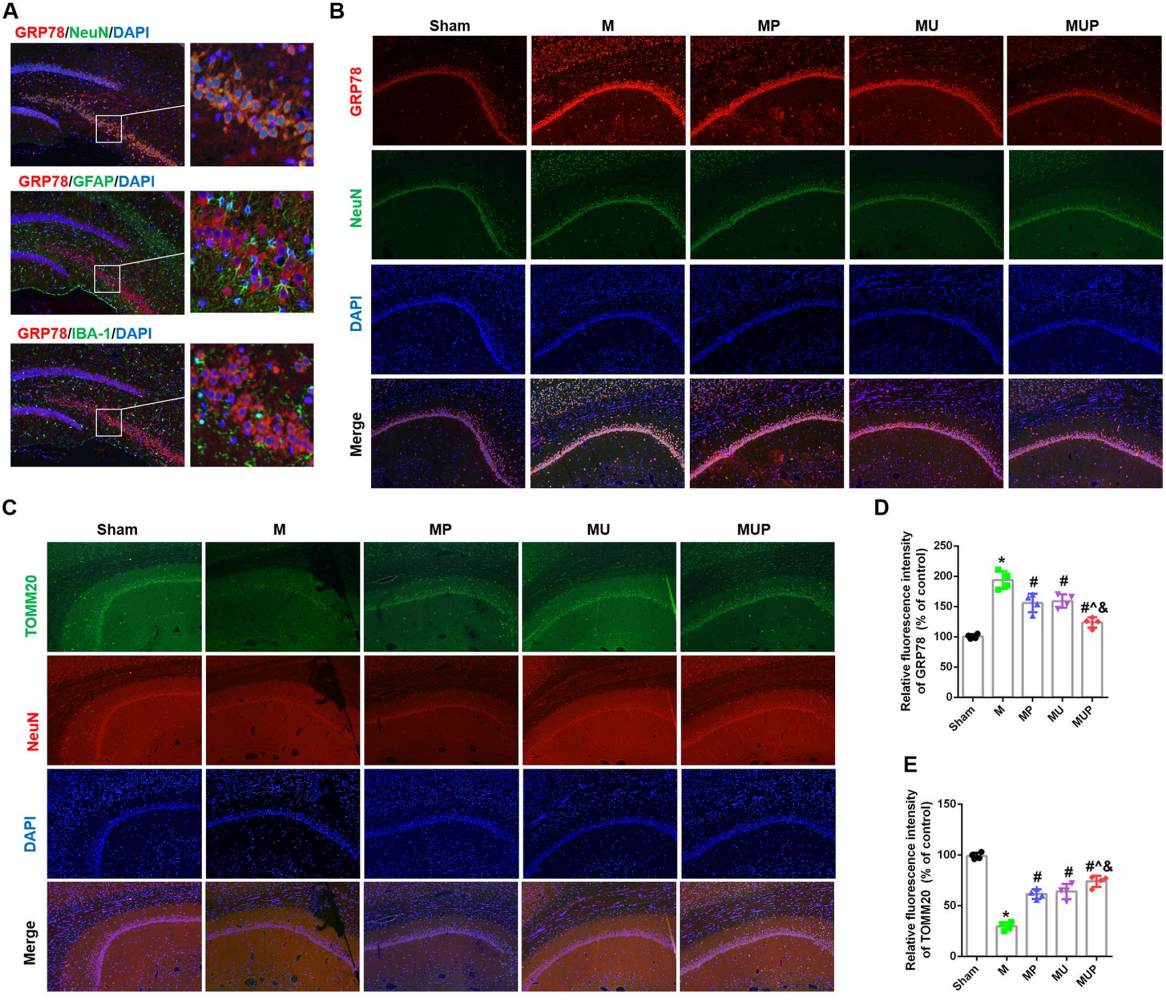
Effects of URB597 and 4-PBA on CCH-induced ER stress and mitochondrial function. **A** Representative double staining between GRP78 (red) and NeuN, Ibal-1, and GFAP (green) in the hippocampus. **B**, **C** Representative GRP78 and TOMM20 double staining with NeuN immunofluorescence, respectively. **D**, **E** Quantitative analysis of the relative fluorescence intensity. ^*^p < 0.05 vs. Sham; ^#^*p* < 0.05 νs. M, ^^^*p* < 0.05 νs. MP, ^&^*p* < 0.05 νs. MU (*n* = 4)

The hippocampus is one of the key brain regions for learning and memory in mammals. Mitochondria and ER are important organelles for the homeostasis and survival of neurons. To expand upon the above findings, the effects of URB597 treatment on ERS and mitochondria in hippocampal neurons in CCH mice were assessed using immunofluorescence staining. First, double staining with GRP78 (red) and different markers of neural cells (green) suggested that a large number of proteins were expressed by neurons (Fig. 6A). Compared with the cerebral ischemia group, URB597 not only inhibited ERS but also protected mitochondria in hippocampus CA1 areas (Fig. 6B, C). A similar trend was also found in the 4-PBA treatment and the co-treatment groups. Statistical analyses suggested that URB597, 4-PBA, and URB597 combined with 4-PBA significantly reversed the ERS and mitochondrial damage caused by CCH (all, *P* < 0.05 vs. M) (Fig. 6D, E). Taken together, in CCH mice, URB597 had the favorable effects of inhibiting ERS and protecting mitochondria.

### The FAAH Inhibitor, URB597, Ameliorates CCH-Induced Ultrastructural Injuries of MAMs

**Fig. 7:**
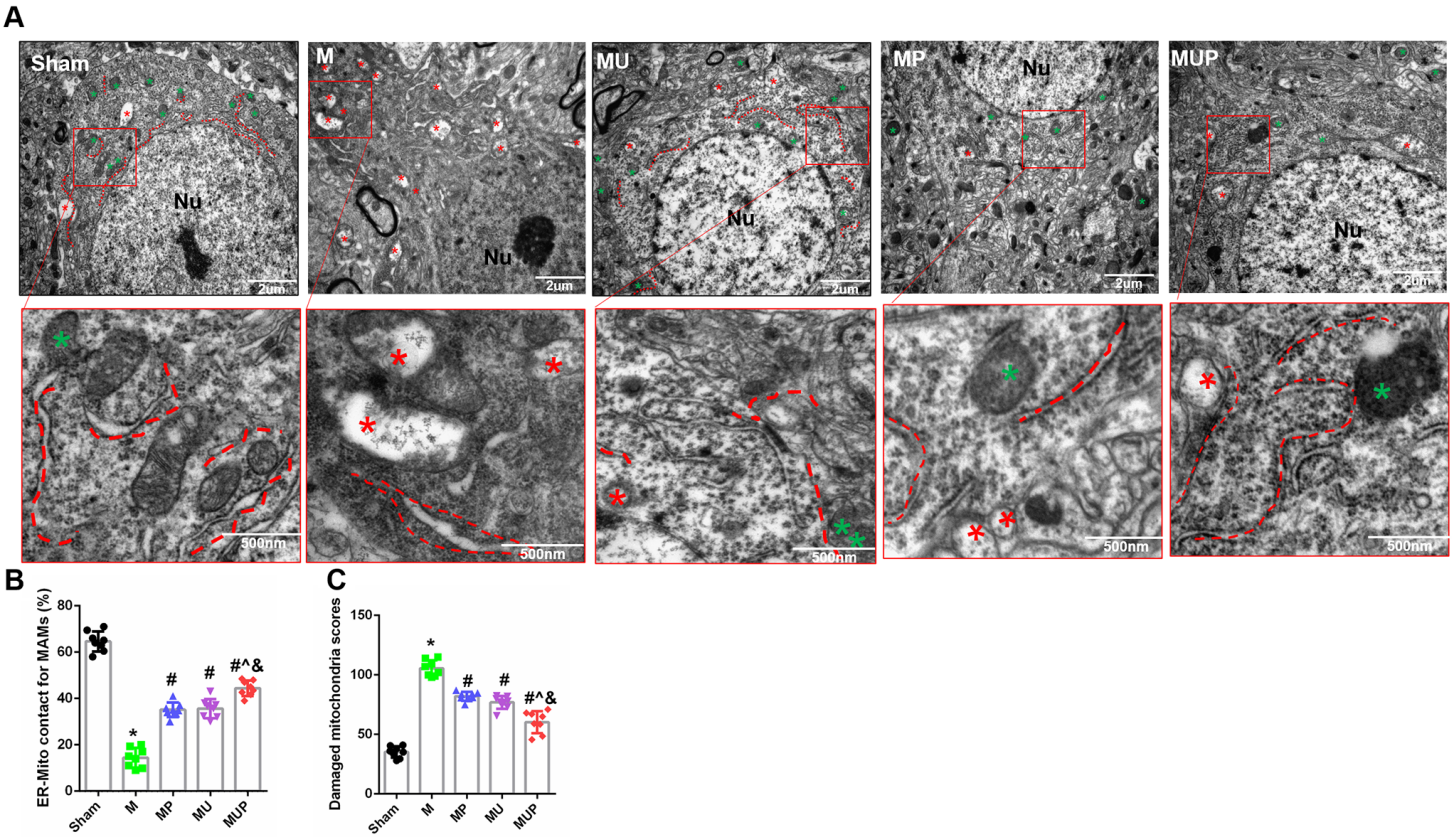
Effects of URB597 and 4-PBA on CCH-induced ultrastructural changes of MAMs. **A** Representative ultrastructure of hippocampal neurons in different groups. Neurons with normal neuron nuclei and the ER and mitochondria in the Sham group were found. There were many coupling MAM regions between mitochondria and ER with normal morphology. Ultrastructural deterioration of mitochondria, the ER, and MAM regions were induced by CCH. The number of organelles decreased significantly. Swollen mitochondria and ER with disrupted MAMs. Ultrastructural changes after chronic treatment with URB and 4-PBA involved reduced degenerated organelles, slightly swollen mitochondria, normal ER, and improved MAMs in structure and quantity. **B**–**C** Quantitative analysis of MAMs and mitochondria injury. Nu: nucleus. Red dotted lines: ER; green asterisk: normal mitochondria; red asterisk: abnormal mitochondria. ^*^p < 0.05 vs. Sham; ^#^*p* < 0.05 νs. M, ^^^*p* < 0.05 νs. MP, ^&^*p* < 0.05 νs. MU (*n* = 4)

MAMs are not only very important for the functions of mitochondria and the ER, but also regulate communication between the two organelles. Ultrastructural changes of mitochondria and the ER were detected by TEM (Fig. 7A). Severely swollen and degenerated mitochondria with disrupted crista and large vacuoles were found in the cytoplasm of ischemia neurons. The ER was expanded and the number of residual bodies increased, while some even disappeared. The gap between mitochondria and the ER widened and disappeared. The apoptotic or degenerated neurons with obscured cytoplasm and reduced MAMs were found in the M group. The number of organelles also decreased significantly after CCH. However, morphological defects of the mitochondria, the ER, and MAMs in mice of the MU, MP, and MUP groups were alleviated, when compared with mice in the M group. Specific criteria of neuronal mitochondria injury were used for a more in-depth analysis using Flameng’s score (Table 1) The score of neuronal injury was significantly increased in the M group (*P* < 0.05, vs. Sham), which decreased after URB597, 4-PBA, and co-treatments, with improved interactions of five MAMs (all, *P* < 0.05, vs. M) (Fig. 7B, C). Together, these results confirmed that URB597 ameliorated MAM impairment induced by CCH.

### The FAAH Inhibitor, URB597, Activates CB2/β-Arrestin1/PERK Signaling

**Fig. 8:**
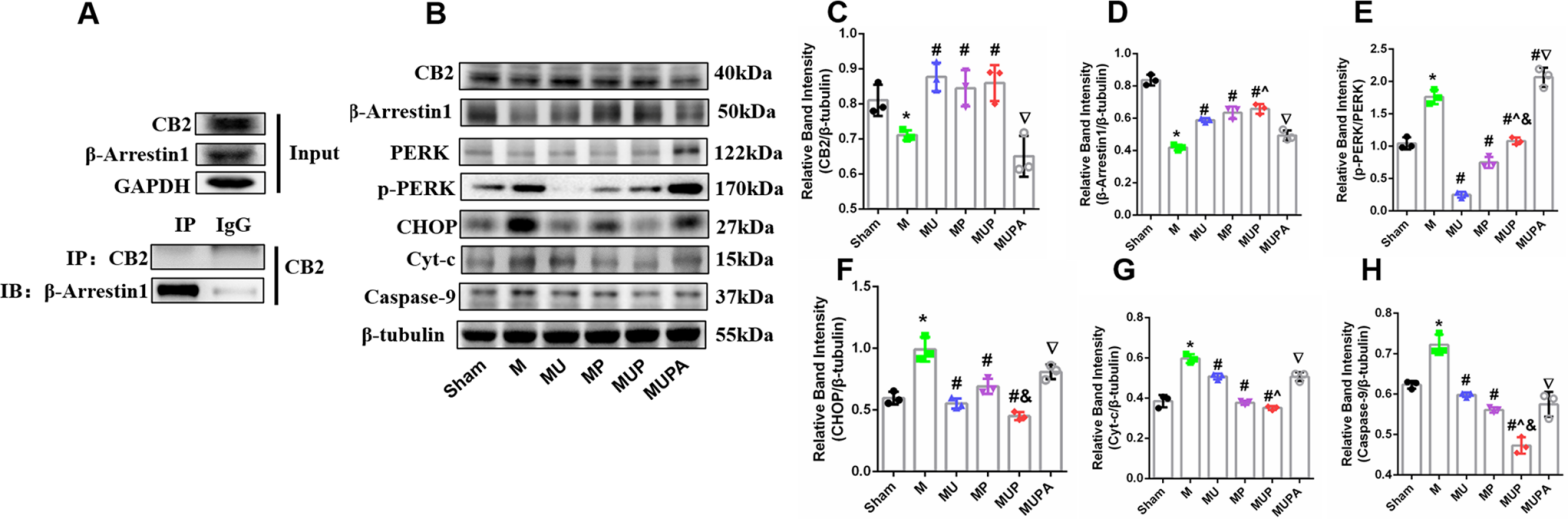
Effects of URB597 and 4-PBA on the CB2/β-Arrestin1-dependent ER stress and mitochondrial apoptosis. **A** Immunoprecipitation confirmed the interaction between CB2 and β-Arrestin1. **B** Representative western blots of CB2, β-Arrestin1, p-PERK/PERK, CHOP, Cyt-c, and caspase-9. **C**–**H** Quantitative analysis of protein expression. ^*^p < 0.05 vs. Sham; ^#^*p* < 0.05 νs. M, ^^^*p* < 0.05 νs. MP, ^&^*p* < 0.05 νs. MU, ^▽^*p* < 0.05 νs. MUP (*n* = 3)

ECS functional activities can be regulated by FAAH. It has been reported that the FAAH inhibitor, URB597, has anti-inflammatory and antioxidant effects during ischemic stroke and traumatic brain injury. The β-arrestin1 has also been reported to be involved in regulating mitochondrial damage and ERS. To clarify the mechanism responsible for the effects of URB597, we determined changes in CB2/β-Arrestin1, ERS, and organelle apoptosis-related proteins. First, co-immunoprecipitation (Co-IP) experiments confirmed the interaction between CB2 and β-Arrestin1 (Fig. 8A). CB2 and β-Arrestin1 protein levels were significantly increased in the URB597, 4-PBA, and co-treatment groups, when compared with those in the M group (*P* < 0.05, vs. M) (Fig. 8B–D). In contrast, the phosphorylation level of PERK (p-PERK) was decreased in mice with CCH (*P* < 0.05, vs. Sham) (Fig. 8B, E). CCH increased the levels of organelle-specific apoptosis-related proteins (CHOP, Cyt-c, and caspase-9), which was decreased by URB597 treatment (*P* < 0.05, vs. M) (Fig. 8B, F–I). Furthermore, in the CB2 antagonist AM630 group, the above effects were partly reversed (*P* < 0.05, vs. MUP), showing a similar trend compared with those in the M group (Fig. 8B, F–I). All of these results indicated that URB597 inhibited ERS and protected mitochondria in the CB2-dependent model.

## Discussion

Cerebral ischemia is a severe form of stress, causing disturbances in most molecular-biological cellular and organelles processes (Paschen and Doutheil 1999). The ER is a complex, dynamic organelle that mediates numerous responses, such as lipid metabolism, Ca^2+^ storage, and protein folding and repair. ER stress is essential to determine the fate of neurons during brain ischemia (Su and Li 2016). Ischemic injury initiates the unfolded protein response (UPR), which is regarded as a protective mechanism (Han et al. 2021). However, an excessive UPR is usually caused by brain trauma, cerebral ischemia-reperfusion, and stroke, leading to irreversible neuronal damage (Guo et al. 2021; Han et al. 2021). In the present study, we found that ER stress was triggered by OGD and was secondary to oxidative stress and mitochondrial damage. Three ER membrane-embedded sensors, GRP78, ATF6, and PERK, were activated by perturbed ER homeostasis. The level of those proteins as well as the phosphorylation of PERK were increased in OGD. In addition, damage to mitochondrial function has also been confirmed with the decrease in TOMM20 fluorescence and ATP. The ER and mitochondria, as key organelles that control intracellular ROS levels, are prone to oxidative stress due to the destruction of their structure and function (Resende et al. 2022). An abnormal increase in ROS and MDA, and a decrease in antioxidant activity (SOD and CAT) indicates that cells are suffering from oxidative stress, which is considered one of the main factors in ischemic cerebral damage and cognitive impairment (Jurcau and Simion 2020; Orellana-Urzua et al. 2020).

URB597 (C_20_H_22_N_2_O_3_) is a highly selective inhibitor of FAAH. It promotes the activities of ECS by enhancing its ligand and receptor. We previously reported the anti-inflammatory and antioxidant effects of URB597 on OGD. URB597 protects primary cultured hippocampal neurons and brain microvascular endothelial cells against OGD-induced oxidative stress and neuroinflammatory injury (Wang et al. 2021b). URB597 also inhibits ischemic cognitive decline by activating CB1/AKT/BDNF signaling in the CCH rat model (Wang et al. 2017a, 2021c). URB597 shows neuroprotective effects on neuropathic pain, addiction, and depression through multiple mechanisms, and prevents dendrite loss, microglia response, and nicotine-dependent behaviors without evoking classical cannabinoid-like effects (e.g. hypothermia, catalepsy, and hyperphagia) (Piomelli et al. 2006; Wang et al. 2017b; Ebrahimi-Ghiri et al. 2021). However, the effects of URB597 on ER stress and the role of ER stress in the process of CCH remain unclear. Here, inhibition of ER stress by 4-PBA and URB597 produced significant neuroprotective effects and was demonstrated in the cerebral ischemia model, both in vivo and in vitro. Water maze test results suggested that inhibition of ER stress improved CCH-induced cognitive impairment. Furthermore, CCH-induced ultrastructural deterioration of ER and mitochondrion, including abundant ER swelling, abnormal mitochondria membrane swelling, and mitochondrial crista rupture, were ameliorated by URB597 treatment. Nissl staining in a single treatment group or co-treatment group also supported these findings. Recently, a study confirmed that drug-targeted restraint of ER stress alleviated CCH-induced synaptic plasticity injury, oxidative stress, and neuronal apoptosis (Thangwong et al. 2022), which is consistent with our findings. In addition, the pH of treatment fluids may affect its efficacy, as the potency of URB597 is pH-dependent (Paylor et al. 2006), which is a very important factor needed to be considered in further studies.

Mitochondria and ER are dynamic organelles that communicate with each other. There are membrane contacts termed MAMs that provide an excellent scaffold for crosstalk between the ER and mitochondria, allowing rapid exchange of biological molecules to maintain cellular health (Missiroli et al. 2018). Dysfunctions in the MAMs are associated with pathological conditions and human diseases, including neurodegenerative diseases, aging, and traumatic brain injury (Veeresh et al. 2019; Markovinovic et al. 2022). In CCH, the disintegration of MAM ultrastructure was verified by TEM in our study. Currently, there is controversy about the changes and roles of MAMs in vascular dementia and Alzheimer’s disease (AD) pathology. Some researchers have found that MAM-localized functions are increased significantly in cellular and animal models of AD (Area-Gomez and Schon 2017; Zhao et al. 2022). However, several studies have now reported that MAMs regulate neuronal health and synaptic transmission that are damaged in patients with cognitive dysfunction (Area-Gomez et al. 2012; Markovinovic et al. 2022). Such a different conclusion has resulted from the different disease models. The cellar/animal model is different from the chronic pathological changes in humans. In addition, highly activated MAMs may be a compensatory mechanism. Although upregulated MAM function and increased ER-mitochondria communications have been confirmed in AD and ischemic brain injury, mitochondria are extremely susceptible to destruction and reduction in aging, AD, and chronic ischemic cerebrovascular disease (Ham and Raju 2017; Wang et al. 2020b), whose amount directly affects the number and range of MAMs.

CB2 was initially regarded as a peripheral immunomodulation receptor since it was discovered in 1993 (Raitio et al. 2005). However, CB2 has been recently shown to be expressed in both glial cells and neurons, and is involved in multiple functions at cellular and behavioral levels (Jordan and Xi 2019). Brain CB2 is inducible and neuroprotective via up-regulation in response to various insults (Jordan and Xi 2019). In the present study, we found that CCH decreased CB2 expression in the hippocampus, which was associated with CCH-induced neuronal injury. Contartese et al. reported that activation of CB2 protected rat brain cortical slices against OGD and reperfusion injury (Contartese et al. 2012). In a spinal cord ischemia-reperfusion rat model, exogenous activation of CB2 using the agonist, JWH-133, attenuated ischemia-induced neurological deficits (Jing et al. 2020). URB597 can promote the expression of CB2 while inhibiting ER stress, and also upregulates the level of β-Arrestin1. In addition, there is a direct interaction between CB2 and β-Arrestin1, which has been confirmed by Co-IP experiments. The β-Arrestin1 not only directly regulates the expression of PERK, but also activates the Nrf2 pathway and reduces oxidative stress (Liu et al. 2019; Tan et al. 2021). The β-Arrestin1 plays a pivotal role in ER stress signaling pathways that have previously been observed in neurons (Sharma et al. 2021). Here, we also showed that upregulation of CB2 by URB597 was significantly reversed by treatment with the CB2 antagonist, AM630. Interestingly, the expression of PERK protein was elevated following AM630 treatment. One of the potential mechanisms was that AM630 may stimulate microglial accumulation, further aggravating inflammatory responses and ER stress (Tang et al. 2015). Another reason was that PERK can be regulated by the p38 MAPK pathway, which is activated to a certain extent by AM630 (Guo et al. 2021). To the best of our knowledge, these results suggested for the first time that inhibition of ER stress and protection of mitochondria by URB597 was CB2/β-Arrestin1 dependent.

## Conclusion

Taken together, these observations suggest novel mechanisms of URB597 and insights into cognitive impairment during ischemic cerebrovascular diseases, and identify CB2 as a potential target for therapy of ischemic cerebrovascular diseases. Thus, a deeper understanding of how CCH accelerates the vascular-mediated hippocampal neuropathology could potentially provide for preventive interventions, which is vital in developing effective treatments to reverse early symptoms and slow cognitive decline.

## Data Availability

Data will be made available upon reasonable request.

## Abbreviations

AD: Alzheimer’s disease
BCCAO: bilateral common carotid artery occlusion
BDNF: brain-derived neurotrophic factor
CAT: catalase
CBR: cannabinoid receptor
CCH: chronic cerebral hypoperfusion
Co-IP: co-immunoprecipitation
CHOP: C/EBP-homologous protein
Cyt-c: cytochrome-c
DHE: dihydroethidium
DMEM: Dulbecco’s Modified Eagle Medium
ECS: endocannabinoid system
ER: endoplasmic reticulum
FAAH: fatty acid amide hydrolase
GFAP: glial fibrillary acidic protein
GRP78: 78-kDa glucose-regulated protein
MDA: malondialdehyde
MAMs: mitochondria-associated ER membranes
MWM: Morris water maze
OD: optical density absorbance
OGD: oxygen-glucose deprivation
PERK: protein kinase R-like ER kinase
ROS: reactive oxygen species
SD: standard deviation
SOD: superoxide dismutase
TEM: transmission electron microscopy
TOMM20: translocase of outer mitochondrial membrane 20
TG: thapsigargin
UPR: unfolded protein response
URB597: 3´-carbamoylbiphenyl-3-yl cyclohexyl carbamate
VaD: vascular dementia
4-PBA: benzenebutyric acid. MU,the BCCAO + URB597 group
MP: the BCCAO + 4-PBA group
MUP: the BCCAO + URB597 + 4-PBA group
MUPA: the BCCAO + URB597 + 4-PBA + AM630 group
